# Peripheral global neglect in high vs. low autistic tendency

**DOI:** 10.3389/fpsyg.2014.00284

**Published:** 2014-04-04

**Authors:** Daniel P. Crewther, David P. Crewther

**Affiliations:** Centre for Human Psychopharmacology, Swinburne University of TechnologyMelbourne, VIC, Australia

**Keywords:** global percept, local percept, diamond illusion, magnocellular, parvocellular, autism, autistic tendency, AQ

## Abstract

In addition to its core social deficits, autism is characterized by altered visual perception, with a preference for local percept in those high in autistic tendency. Here, the balance of global vs. local percepts for the perceptually rivalrous diamond illusion was assessed between groups scoring high and low on the Autism Spectrum Quotient (AQ). The global percept of a diamond shape oscillating horizontally behind three occluders can as easily be interpreted as the local percept of four line elements, each moving vertically. Increasing the luminance contrast of the occluders with respect to background resulted in an increase of initial global percept in both groups, with no difference in sensitivity between groups. Presenting the target further into the periphery resulted in a marked increase in the percentage of global perception with visual field eccentricity. However, while the performance for centrally presented diamond targets was not different between AQ groups, the peripheral global performance of the High AQ group was significantly reduced compared with the Low AQ group. On the basis of other imaging studies, this peripheral but not foveal global perceptual neglect may indicate an abnormal interaction between striate cortex and the Lateral Occipital Complex (LOC), or to differences in the deployment of attention between the two groups.

## Introduction

Autism (American Psychiatric Association, 2000) and Autism Spectrum Disorder (ASD), as clinically diagnosed (American Psychiatric Association, 2013), are characterized by impaired social communication and interaction and also by restricted or inflexible and often repetitive behaviors. However, typically, there are perceptual differences between autistic individuals and controls without autism (Plaisted et al., 1998a,b; O'Riordan et al., 2001). Children and adults with high functioning ASD have been reported to show superior performance in the recognition of hidden figures, attention to detail in visual search, and in the discrimination of novel stimuli (Plaisted et al., 1998a,b) compared with controls. However, this is at the expense of certain motion sensitivities and global or gestalt perception (Happe and Frith, 2006). Altered visual perception is supported by fMRI studies reporting significantly different activation within the occipital and temporal regions of autistic individuals (Pelphrey et al., 2007; Solomon et al., 2009) when compared to the normal population. Dakin and Frith (2005), in review, concluded that the underlying neural basis of reduced visual motion perception in autism is not explained by a general magnocellular deficit, but is localized to the higher order motion processing area of the Superior Temporal Sulcus (STS) rather than early cortical processing areas such as Area MT/V5. They based their conclusions on a poorer second order motion sensitivity motion defined by progressive contrast change rather than luminance change: (Bertone et al., 2003, 2005) and on mixed results regarding simple first order motion coherence threshold deficits (Spencer et al., 2000; Milne et al., 2002; Bertone et al., 2003) and unimpaired flicker contrast sensitivity (Pellicano et al., 2005).

Two major cognitive theories have emerged to explain the underlying neural basis of differences in perceptual function of individuals with high functioning autism. The Weak Central Coherence (WCC) model of autism (Frith and Happe, 1994) originally proposed a core deficit in central processing resulting in failure to extract global form or meaning. In seeking a physiological explanation, Spencer et al. (2000) and Milne et al. (2002), on the basis of impaired motion coherence thresholds, argued that the dorsal visual cortical stream is weaker in autism than in normals. The superior local processing in autism led to the Enhanced Perceptual Function (EPF) hypothesis (reviewed, Mottron et al., 2006), whereby overdevelopment of low-level perceptual operations related to detection and discrimination were related to an enhanced function of low level visual cortical areas in autism. A more recent version of the WCC (Happe and Frith, 2006) has seen a convergence of the two hypotheses.

Behavioral characteristics of autism exist in all individuals, to a greater or lesser extent. Baron-Cohen et al. (2001) developed a scale—the Autism Spectrum Quotient (AQ), which measures autistic tendency in individuals with IQ in the normal range. AQ scores (median around 15/50 for a neurotypical population, Almeida et al., 2010) appear to smoothly merge from neurotypical to clinical populations with a cross-over point of around 32 (Baron-Cohen et al., 2001) more recently refined to around 26 (Woodbury-Smith et al., 2005). Even so, in an MRI study demonstrating structural discriminants for autistic vs. normal brains (Ecker et al., 2010), the clinically autistic population included individuals with AQ scores as low as 20 and controls with AQ scores over 30.

Individuals with high functioning autism have been found to be capable of attending to global form, but limit their attention selectively (Plaisted et al., 1999), tending to a local attentional state when not goal-directed. While Edgin and Pennington (2005) found benefits in embedded figures reaction times without spatial deficit for high functioning ASD vs. control populations, most other evidence points to some form of deficits in global aspects of attention (Rinehart et al., 2000; Milne et al., 2002; Sutherland and Crewther, 2010). Evidence of deficits in motion perception is somewhat more variable (Bertone et al., 2003, 2005; Pellicano et al., 2005; Sutherland and Crewther, 2010). Differences in global percept between those with high and low autistic tendency are particularly noticeable in the performance on biological motion tasks (Rutherford and Troje, 2012; Van Boxtel and Lu, 2013).

Direct physiological evidence for the status of the magnocellular system in autistic tendency derives from EEG and particularly from nonlinear VEP using Wiener kernel analysis and m-sequence pseudorandom stimulation (Sutter, 2000). Thus, (Klistorner et al., 1997) showed that in a neurotypical population, the second order kernel peaks largely separate into magno and parvocellular generated non-linearities on the basis of recovery time, with responses categorized on the basis of contrast gain, saturation and latency. Sutherland and Crewther (Sutherland and Crewther, 2010) showed that a group high in autistic tendency showed reduced low temporal contrast first order kernel response for the initial negative response (N50) compared with a group low in autistic tendency. Significantly impaired magnocellular recovery after stimulation was reported in a High AQ group compared with Mid and Low AQ groups (Jackson et al., 2013).

**Figure 1:**
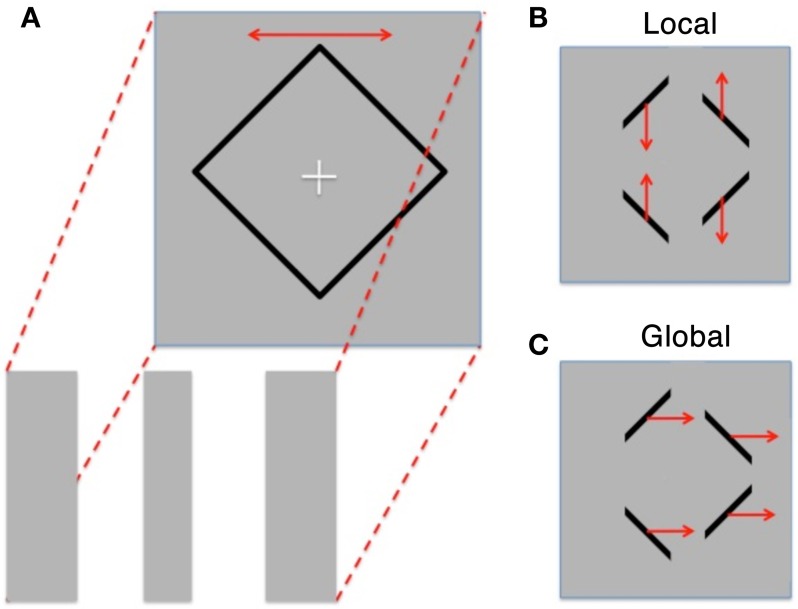
**(A)** Schematic of the diamond illusion showing the diamond shape oscillating horizontally behind three occluding stripes of the same color and luminance as the background. **(B)** Resultant perception is either “Local” with 4 lines moving up and down or **(C)**. “Global” perception where the motion of the lines is grouped and they are seen as moving horizontally back and forth (redrawn from Fang et al., 2008).

We aimed to explore differences in global/local perception within High and Low AQ individuals using the Diamond Illusion (Lorenceau and Shiffrar, 1992). In this perceptual rivalry stimulus, observers view either a diamond shape sliding horizontally behind three occluders, or instead see four line segments oscillating vertically (see Figure 1). Alternation between the two percepts occurs after an initial percept is established.

Fang, Kersten and Murray (Fang et al., 2008) used fMRI techniques to explore brain activation when global and local percepts were dominant. They showed a reciprocal activation between the object encoding lateral occipital complex (LOC) and primary visual cortex (V1), with LOC activation increasing and V1 activation reducing, when a switch from local to global percept is reported, and *vice versa*, confirming earlier reports of reduced occipital cortical activation when shape is observed in patterned stimulation (Murray et al., 2002, 2004).

We postulated that if those with High AQ possess a less automatic global perceptual system when confronted with an ambiguous stimulus such as the diamond illusion, then initial local percepts should occur relatively more frequently. Two experiments were planned. In the first, we examined the effect of contrast between occluding stripes and background on the initial percept (global or local) for High and Low AQ groups. The second experiment derives from an observation of (Lorenceau and Shiffrar, 1992), that the diamond or global form was easier to observe when presented 7° peripherally, although Lorenceau and Shiffrar did not explore this phenomenon to a great degree, merely attributing it to the larger dimensions of receptive fields at peripheral locations. We proposed, under repeated trial conditions at different stimulus eccentricities, that those high on the AQ scale would show a reduced percentage of initial global percepts compared with those with Low AQ, especially in the periphery. Supplementary Figure 1 shows a movie demonstrating this phenomenon—as fixation moves away from the diamond target, percept typically switches from local to global.

## Materials and methods

### Participants

Fifty four neurotypical individuals (28 female, 24 male and 2 unreported; age range 18–37 years, *M* = 25.74, *SD* = 4.90) took part in the study. The participants were selected from a total population of 290 directed through flyers, emails and social networking websites to an online survey tool (Opinio, www.objectplanet.com/opinio/). Following the reading of the approved information for participants page explaining the project in full, participants volunteered by clicking the accept button and were presented with an online version of the AQ (Baron-Cohen et al., 2001). The AQ is a scale of 50 questions related to social and environmental lifestyle choices. Participants answered questions such as “I know how to tell if someone listening to me is getting bored,” or “New situations make me anxious” via a 4-point Likert scale (definitely agree to definitely disagree), in radio button format using an on-line survey tool. Following the online completion of the AQ and a short demographic survey (age, gender, education and occupation), a High AQ group with AQ scores >19 (mean = 24.4, *SD* = 4.1, *n* = 21) and a Low AQ group with scores <11 (mean = 7.7, *SD* = 2.1, *n* = 15) were invited by email to participate in further psychophysical testing. Participants also completed the 21 item version of the Depression, Anxiety and Stress Score (Henry and Crawford, 2005), and the Ravens Advanced Progressive Matrices Test (Raven, 1998), however this information was not included as part of the current analysis.

The study was approved by the Swinburne University Human Research Ethics Committee and complied with the provisions of the Treaty of Helsinki. Participants were free to withdraw from the study at any point during the testing period.

### Occluder contrast task

Participants were initially introduced to the Diamond illusion through the presentation of the stimulus with high contrast occluders and with zero contrast occluders—each for 30 s, making the global and local percepts highly visible. The experimental protocols continued after confirmation from the participants that both possible perceptions had been clearly seen.

All experimental conditions were programmed and presented using VPixx (VPixx Technologies, v.2.14). An initial diamond (11 × 11°) was presented in black on a uniform gray background (contrast 92%) on a computer screen (MacBook Pro, 17”) to the participant. The diamond outline shape oscillated horizontally (amplitude 3°, frequency 0.5 Hz) under three rectangular occluders which obscured all vertices of the diamond (see Figure 1). These occluders either had the same luminance as the background, or were darker than background (with Michelson contrasts of 0.25, 0.5, 0.75, 1.0, 1.25, 1.5, or 2.0%). Each condition was repeated five times and participants were asked to indicate in a 2AFC (two alternate forced choice) fashion, when the stimulus appeared abruptly around a central fixation cross, whether their initial percept was global or local. Response was indicated by pushing buttons corresponding to the motion direction observed (vertical or horizontal).

### Diamond periphery task

In the Diamond Periphery Task, a fixation cross was presented in either the left or right region of the screen. Participants were requested to fixate the cross, and after a 1 s delay, the stimulus (subtending 5.5 × 5.5° and oscillating horizontally with amplitude 1.5°, frequency 0.5 Hz), was presented at varying eccentricities (0, 8, 11.5, 14, 16.5, and 19°) in right and left visual hemifields for a period of 2.5 s after which the stimulus disappeared and the next trial did not start until a response (vertical or horizontal) had been given. The stimuli were viewed at a distance of 57 cm. Mean background luminance of the screen (gray) was 50 cd/m^2^, and the occluders were the same luminance as the background. The task was scored in terms of the proportion of initial percepts seen as global rather than local.

## Results

### Occluder contrast task

**Figure 2:**
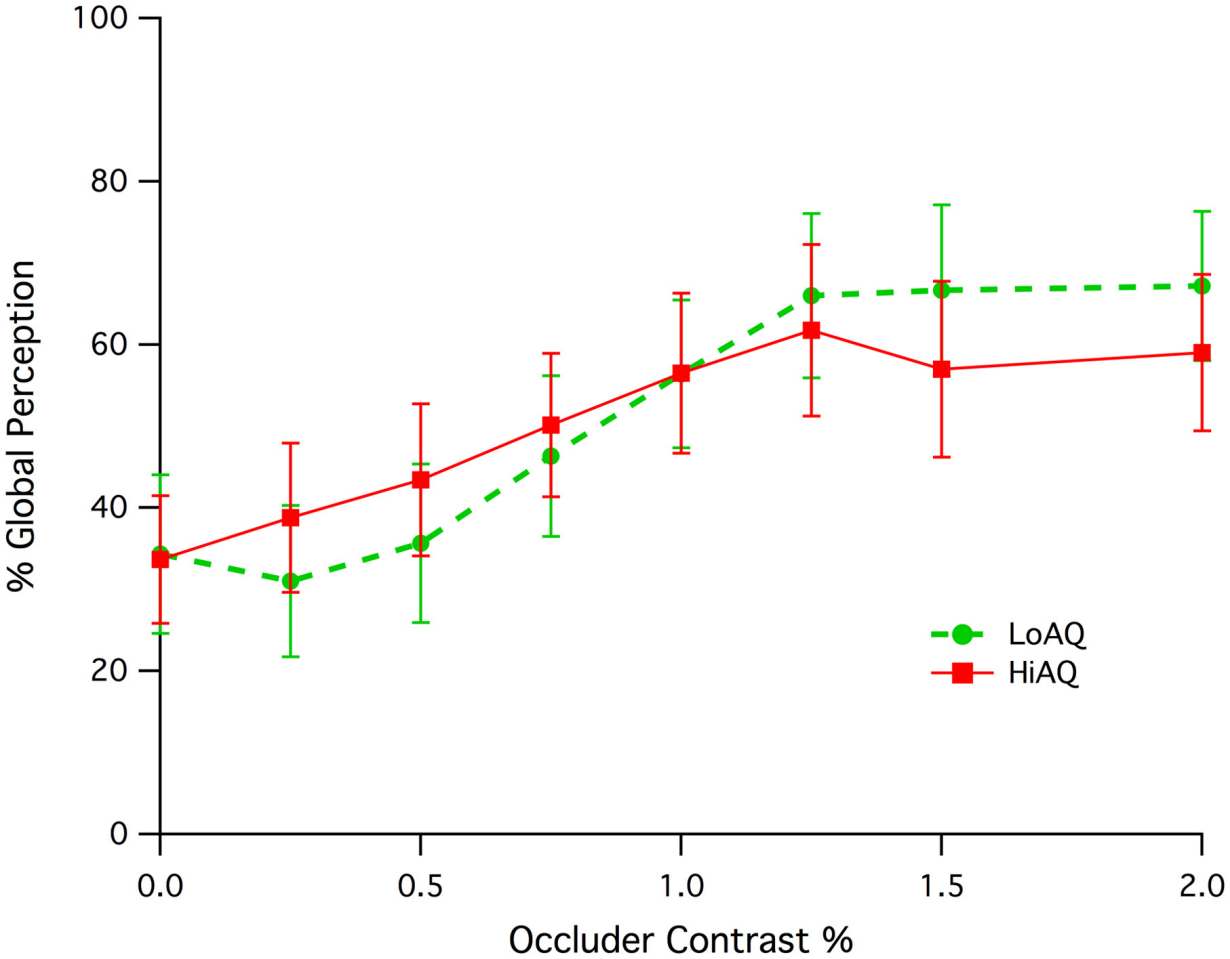
**Means and standard error of initial global percept (expressed as a percentage) as a function of occluder contrast for the Low and High AQ groups**.

Both High AQ and Low AQ groups showed an increase in initial global percept as the occluding screen was more readily revealed through luminance contrast with respect to the background (see Figure 2). Both groups appeared to reach a plateau of global perception at a contrast of around 1.25%. A significant correlation of percent global percept vs. occluder contrast for the combination of High and Low AQ groups between contrasts of 0.0 and 1.0% was found (Pearson, *r* = 0.185, *df* = 142, *p* = 0.015). There was no statistically significant difference between groups, despite the apparent difference in slope. Permutation testing (developed using LabView) on the correlations for High and Low AQ groups between contrasts of 0.25 and 1.0% gave a probability of difference in correlation of *p* = 0.116 indicating a lack of significant difference.

### Diamond periphery task

**Figure 3:**
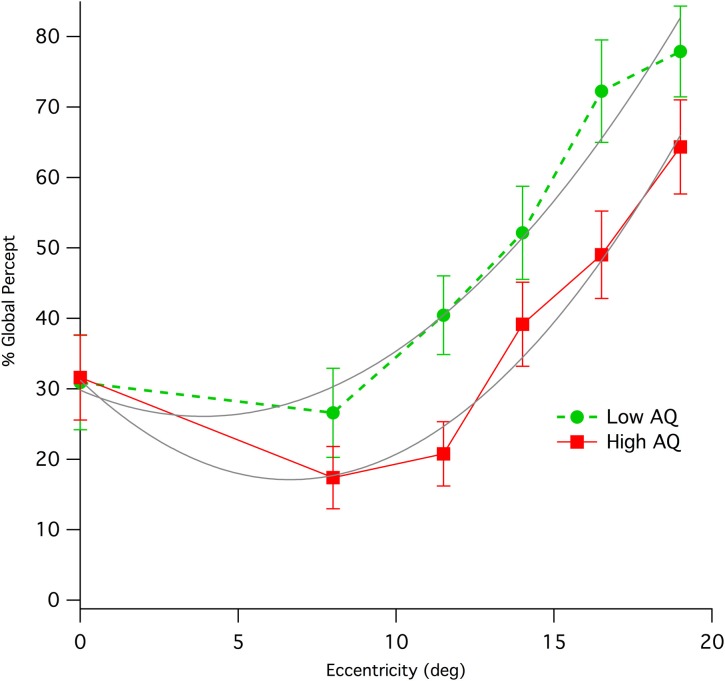
**Means (with standard error) of percentage of initial global percept for Low and High AQ groups when viewing the stimulus at various eccentricities in visual field**. The data for High AQ are shown with a solid red line, while those for the Low AQ group are shown as a dashed green line. Parabolic fits (gray color) to the two sets of data allow a comparison of relative eccentricities required to give equivalence of global percept between the High AQ and Low AQ groups.

Results from left and right peripheral viewing of the diamond illusion were combined due to the small trial numbers. Initial global percentage percept shows an immediate and strong effect of eccentricity, increasing rapidly with up to eccentricities of 19° (see Figure 3), where individual data show some evidence of ceiling effects. Collapsing data from the two AQ classes and from both hemifields of presentation shows a strong correlation of global percept with eccentricity (Pearson, *r* = 0.389, *df* = 248, *p* < 0.0001).

Inspection of Figure 3 shows a distinct difference between AQ groups. While performance foveally (ie for central fixation) was not different between groups, peripheral global initial percept was lower for the High AQ group compared with Low AQ. Parabolic fits to the data using the Levenberg-Marquardt least-squares method implemented in IGOR Pro (Wavemetrics) were carried out (LowAQ: Global% = 29.81 − 1.9*x* + 0.25*x*^2^; HighAQ: Global% = 31.24 − 4.25*x* + 0.32*x*^2^, where *x* is eccentricity in deg). These curve fits demonstrate that, apart from central viewing, in order to obtain equivalent levels of global perception, those with High AQ have to use peripheral viewing of at least 10° eccentricity. Repeated measures ANOVAs on the three mid-peripheral eccentricities (11.5, 14, 16.5°) showed a significant between group effect [*F*_(1, 66)_ = 4.42, *p* = 0.039]. The most extreme peripheral data were not included because of the likelihood of ceiling effects in some of the Low AQ participants at this eccentricity. We tested for effects of hemisphere of presentation, however there was no main effect. However, there was a marginal interaction [Hemifield ^*^ Eccentricity ^*^ AQ group: Greenhouse-Geisser *F*_(2.84, 85.1)_ = 2.55, *p* = 0.064], with some sign of greater deficit in global percept for the High AQ group from presentation in left hemifield.

## Discussion

This study explored two aspects of visual perception for ambiguous global and local stimuli using groups of young adults with High and Low AQ scores. Rather than giving a defined attentional goal in terms of global or local percept, the first experiment aimed to evaluate the prevalence of initial global or local percept when the potential meaning of the stimuli is biased by making the presence of occlusion more obvious (through increasing contrast with respect to the background). While the results demonstrated a significant relationship between the Michelson contrast of the occluders and percent global percept, they also show that High AQ and Low AQ groups perform in a very similar fashion. This suggests that the presence of occlusion is revealed to the same degree in both groups and suggests a commonality of low level contrast sensitivity for edges, ie, the perception of low contrast objects is not different between the two groups in terms of foreground/background segmentation.

However, significant differences were found in the global/local sensitivity when the rivaling stimuli are viewed peripherally. For both High and Low AQ groups, increasing retinal eccentricity significantly increased the percentage of trials in which the global percept was first seen. However, the results was not a simple superiority in global perception for Low vs. High AQ group, as the foveal performance was nearly identical between groups. Rather, as the target was presented more peripherally, its likelihood of being initially identified as the Global form was markedly impaired in the High AQ group compared with the Low AQ group.

Although we confirmed Lorenceau and Shiffrar's (1992) observation that the diamond (global percept) is more easily visualized at 7° in the periphery, their explanation, based around motion integration, would predict greater ease of global percept with eccentricity due to larger receptive fields. While both groups showed an increase in global percent percept in the periphery, and while population Receptive Field (pRF) size estimates using fMRI techniques (Zuiderbaan et al., 2012) in autism (Schwarzkopf et al., 2014) report larger receptive fields for those with high functioning ASD compared with controls at any eccentricity, these between group differences are in the wrong direction to explain the Global perceptual differences. Indeed, larger pRFs for High AQ *cf* Low AQ individuals would predict better rather than poorer global percept in the periphery. The mechanisms proffered for pRF size change included either extrastriate cortical hyperexcitability or differences in attentional deployment, though neither was measured. While poorer functioning of low spatial frequency channels in those with High AQ was been proposed as an explanation of impaired global perception, it seems that the global vs. local nature of the diamond illusion contains another element - probably border ownership (Zhou et al., 2000), that far outweighs the effects of, say blurring, to filter out high spatial frequencies, as viewing the movie in Supplementary Figure 1 under conditions of optical blur easily demonstrates. fMRI studies indicate that area V2, lying between V1 and object activation in LOC, plays a pivotal role in border ownership in human (Fang et al., 2009).

Studies on global precedence of Navon figures (Amirkhiabani and Lovegrove, 1999) explored this size/periphery element, finding that speed of processing at the local level increased when stimuli were displayed foveally, but slowed at peripheral visual field locations—possibly indicating a decrease in local stimulus saliency in the periphery. This presumably is relevant to the periphery task, as a precedence in time should translate into the percept seen first. The higher prevalence of initial local percepts observed for the High *cf* Low AQ group in the periphery could thus be interpreted as a “global peripheral neglect.” The source of the neglect could lie in group functional differences between in connections of V1 and LOC, or by differences in attentional deployment, as suggested above. However, the similarity of global percent percept when viewing is foveal, suggests that reciprocal V1↔LOC activations such as those reported by Fang et al. (2008) under local↔global percept report is likely to occur in both groups. In the current task, the randomization of stimulus location would suggest an exogenous attentional demand, although the limited number of eccentricities (5) probably generates some endogenous sharpening of attentional focus around these locations, ignoring superior and inferior visual field locations.

The question remains as to whether such a peripheral neglect in terms of global perception may result in altered gross behavior, possibly including some of the core symptoms presenting in ASD. According to the DSM-IV criteria (American Psychiatric Association, 2000) it is common for individuals with autism to demonstrate an averted gaze, primarily focusing upon eccentricities when interacting in a social manner. While these face aversions are often interpreted in terms of an avoidance due to a fear response, our results suggest that these behaviors may also represent an automatic attempt by individuals high on the spectrum to obtain better global perception using the global dominance demonstrated in the periphery, to make up for impaired global recognition (Sutherland and Crewther, 2010). The results presented here demonstrate that equivalent levels of global perception require eccentric viewing of at least 10°. Thus, a typically developing child inspecting a face at a distance of a meter or so might use eccentricities out to 5° without incurring a diagnosis of gaze aversion, while still receiving a holistic image. On the other hand, if a similar degree of global percept is sought, the child with ASD would need to be fixating eccentrically by at least an extra 10° which might be interpreted as averted gaze. Gaze aversion has been associated with cognitive load as a management strategy (Doherty-Sneddon et al., 2012), children with ASD showing elevated levels of gaze aversion while listening to questions, but not while making their responses. Where direct psychophysical measurement of face processing in normal adults has been made, peripherally presented low spatial frequency information has been shown to enable efficient processing of faces (Awasthi et al., 2011).

Our research suggests that studies of covert attentional focus in ASD and autistic tendency are warranted. If such a use of peripheral covert attention to improve global awareness is being employed by children with ASD, then it is questionable whether they should be trained to look directly at people in social situations without first attempting to train better global perception.

### Conflict of interest statement

The authors declare that the research was conducted in the absence of any commercial or financial relationships that could be construed as a potential conflict of interest.
